# Effects of Dietary *Peganum harmala* Stem Ethanolic Extract on Serum Inflammatory and Antioxidant Markers, Duodenal Metabolomic Features, and Hepatic Transcriptomic Profiles in Hu Sheep

**DOI:** 10.3390/metabo16080591

**Published:** 2026-08-19

**Authors:** Bo Wang, Cunxi Nie, Yanfeng Liu, Rongyan Qin, Wenqi Wang, Yanfen Cheng

**Affiliations:** 1 College of Animal Science and Technology, Shihezi University, Shihezi 832003, China; a719766823@163.com; 2 Xinjiang Key Laboratory of Herbivorous Livestock Feed Biotechnology, Feed Research Institute, Xinjiang Uygur Autonomous Region Academy of Animal Science, Urumqi 830011, China; xjslsqry@163.com (R.Q.); xjslswwq@163.com (W.W.); 3 College of Animal Science and Technology, Nanjing Agricultural University, Nanjing 210095, China; yanfencheng@njau.edu.cn

**Keywords:** *Peganum harmala*, stem ethanolic extract, Hu sheep, inflammatory markers, antioxidant markers, metabolomics, transcriptomics

## Abstract

**Background:** *Peganum harmala* L. stem ethanolic extract (PHL) has been scarcely evaluated as a ruminant feed component. We evaluated its effects on serum inflammatory and antioxidant biomarkers and duodenal and hepatic molecular responses in Hu sheep. **Methods:** Twelve male Hu sheep, approximately 120 days old, were randomly assigned to a control group or a PHL treatment group (*n* = 6 per group). After a 7-day adaptation period, the PHL group received 3 g/head/day of PHL (total flavonoid content: 65.7 mg rutin equivalents (RE)/g; approximately 197.1 mg RE/head/day) for 50 days. Serum markers, duodenal untargeted liquid chromatography–mass spectrometry (LC–MS) metabolomics, liver RNA sequencing, and alternative splicing were analyzed. **Results:** Serum interleukin-6 and malondialdehyde were lower, whereas interleukin-4 and glutathione peroxidase were higher, in the PHL group than in the control group (*p* < 0.05). Using variable importance in projection ≥ 1, fold change ≥ 1.2 or ≤0.83, and unadjusted *p* < 0.05 as screening criteria, 479 LC–MS features met these nominal screening criteria, of which 81 had database annotations. Following Benjamini–Hochberg correction, only two features remained significant (q < 0.05); however, only one was successfully annotated, rendering the pathway analysis exploratory. Liver RNA sequencing identified 279 differentially expressed genes (absolute log_2_FC ≥ 1 and q ≤ 0.001) and 104 skipped-exon events (false discovery rate < 0.05). Functional enrichment analysis highlighted annotations related to bile secretion, ATP-binding cassette transport, nutrient metabolism, and glutathione metabolism. **Conclusions:** PHL supplementation was associated with selective changes in serum inflammatory and antioxidant biomarkers, accompanied by hepatic molecular responses. The duodenal metabolomic findings remain exploratory, and dose–response studies incorporating alkaloid quantification and targeted molecular validation are required before practical application can be considered.

## 1. Introduction

Interest in phytogenic feed additives has increased as livestock production systems seek alternatives to antibiotics to support animal health and productivity [1,2,3]. Flavonoids comprise a structurally diverse class of plant secondary metabolites and do not exhibit biologically uniform activity. Many flavonoids exhibit antioxidant and anti-inflammatory properties by scavenging reactive species or modulating inflammatory signaling pathways; however, their biological effects depend on their chemical composition, dose, bioavailability, and the target animal species [4,5].

*Peganum harmala* L. is a drought-tolerant plant distributed in the arid and semi-arid regions of Xinjiang. Its extracts have shown antioxidant, anti-inflammatory, antimicrobial, and metabolic activities in experimental models [6,7,8,9]. However, most research has focused on seeds, β-carboline alkaloids, or crude extracts [6,7]. Because the seed fraction is particularly associated with alkaloids and potential neurotoxicity, stems were selected to avoid deliberate inclusion of alkaloid-rich seed material. The choice of stems does not demonstrate that the stem extract was alkaloid-free, as harmine, harmaline, and other alkaloids were not quantified in the feed batch.

In ruminants, dietary flavonoids can be transformed by gastrointestinal microorganisms and may influence nutrient use, microbial metabolism, host lipid metabolism, and redox balance [10,11,12,13,14]. Meta-analyses and controlled trials of flavonoid and related phytogenic preparations have demonstrated variable, context-dependent effects on performance, digestibility, rumen fermentation, and antioxidant status [15,16,17,18,19]. Evidence regarding the use of *P. harmala* stem ethanolic extract in sheep remains scarce, and the associated intestinal and hepatic molecular responses have not been characterized.

It was hypothesized that dietary *P. harmala* stem ethanolic extract (PHL) would alter inflammatory and antioxidant status and would be accompanied by measurable duodenal and hepatic molecular responses in Hu sheep. Accordingly, this study evaluated growth performance and serum biochemical, inflammatory, and antioxidant markers; quantified duodenal histomorphometry; profiled duodenal-content metabolomic features; and characterized hepatic gene expression and alternative splicing responses following 50 days of supplementation.

## 2. Materials and Methods

### 2.1. Source and Preparation of PHL

Cultivated *P. harmala* stems were collected from Karamay, Xinjiang, China, in September 2024 at an immature phenological stage, approximately two months before the expected full-maturity stage. Because exact sampling-site coordinates were not recorded, the reference coordinate of Karamay city (approximately 45°37′ N, 84°50′ E) is provided only for regional context. The plant material was authenticated morphologically by Yanfeng Liu, and a voucher specimen was retained by the research team under voucher number LTP001. After shade-drying for 1 week, the stems were ground through a 400-mesh screen with an AM300S cutting grinder (Ants Scientific Instruments, Beijing, China). The ground stems were extracted once with 85% aqueous ethanol at a solid-to-liquid ratio of 1:20 (*w*/*v*) for 60 min at 50 °C in a KQ-500DE ultrasonic unit (80 kHz, 500 W; Kunshan Ultrasonic Instruments Co., Ltd., Kunshan, China). After centrifugation at 1600 rpm for 20 min in an 80-2 centrifuge (Guohua, Jiangsu, China), the supernatant was vacuum-dried. Total flavonoid content was 10.67 mg rutin equivalents (RE)/g dried stem material and 65.7 mg RE/g of vacuum-dried concentrated extract, as determined by the rutin–NaNO_2_–Al(NO_3_)_3_ colorimetric method at 510 nm with a UV-1800 spectrophotometer (Shimadzu Enterprise Management Co., Ltd., Shanghai, China). The concentrated extract showed a 6.16-fold enrichment in total flavonoids relative to the dried stem raw material. The extraction conditions were selected on the basis of preliminary optimization work conducted by the authors. The administered dose was 3 g extract/head/day, providing a total-flavonoid dose of approximately 197.1 mg RE/head/day and thereby approximating the planned target of 200 mg RE/head/day. Exploratory untargeted LC–MS profiling performed during extraction optimization tentatively annotated several flavonoids (including santin, kaempferol, lonicerin, and a methoxylated flavonoid feature) and non-flavonoid constituents (including harmine, tetrahydroharmol, deoxypeganine, vasicinone, 4(3H)-quinazolinone, and several phenolic acids). These annotations were based on spectral-library matching and were not confirmed using authentic standards; therefore, absolute concentrations were not determined. Accordingly, the treatment material is described as a stem ethanolic extract rather than a purified flavonoid fraction. PHL is retained as the prespecified treatment code for consistency with the study records and figures.

### 2.2. Animals, Diets, and Experimental Design

The feeding trial was conducted at Huocheng Anxin Husbandry Farm in Huocheng County, Ili Kazakh Autonomous Prefecture, Xinjiang, China. Because farm-level GPS coordinates and elevation were not recorded, the county-seat reference location (approximately 44.05° N, 80.87° E; 637 m above sea level) is reported only for geographical context. Huocheng has a temperate semi-arid climate, with a long-term mean annual temperature of 8.2–9.4 °C and annual precipitation of 140–460 mm.

Twelve healthy male Hu sheep (approximately 120 days old with an average body weight of 33.17 ± 1.41 kg) were obtained from Huocheng Anxin Husbandry Farm and were randomly assigned using computer software to either the control (CON) or PHL group (*n* = 6 per group). Before the adaptation period, all sheep underwent a farm-standard veterinary health examination and quarantine, deworming, routine vaccination, and disinfection of the pens. All sheep underwent a 7-day adaptation period followed by a 50-day feeding period, after which they were slaughtered for sample collection. The CON group received the basal complete pelleted diet, whereas the PHL group received the same diet supplemented with 3 g of vacuum-dried stem ethanolic extract per head per day, providing a total-flavonoid dose of approximately 197.1 mg RE/head/day and approximating the planned target of 200 mg RE/head/day. This single dose was selected with indirect reference to evidence from ruminant plant and flavonoid studies [15,16,17,18,19] and has not been validated specifically for *P. harmala*. The ingredient composition and nutrient profile of the basal diet are presented in Table 1. Dry-matter content was determined from the as-fed diet, whereas metabolizable energy and all other nutrient contents were calculated and expressed on a dry-matter basis.

Sheep were housed individually in naturally ventilated outdoor pens (1.5 × 3.0 m) with perforated plastic slatted flooring. During the trial, ambient temperature and relative humidity ranged between 10–16 °C and 40–60%, respectively. Pens were cleaned once daily. Sheep were fed twice daily at 10:00 and 18:00 and had free access to water; no additional forage was offered. Each daily dose of PHL was mixed thoroughly with a small portion of the pelleted diet and offered first to ensure complete consumption, after which the remaining feed was provided. The CON group received the basal diet without a supplement. The quantities of feed offered and refused were weighed daily for each sheep. The initial feed allowance was 1200 g/day and was increased by 100 g when no refusal was observed or decreased by 100 g when refusals exceeded 10% of the amount offered, with a target refusal rate of approximately 10%. Animals were observed daily. No refusal of the administered dose, signs of disease, abnormal behavior, or mortality were observed throughout this study.

### 2.3. Growth Performance and Serum Measurements

Body weight was measured at 11:00 on days 1 and 50 after feed had been withdrawn for 24 h; water remained available throughout the fasting period. Average daily gain (ADG) was calculated as (final body weight − initial body weight)/50. Average daily feed intake (ADFI) was calculated for each sheep as the mean daily difference between the amounts of feed offered and refused on an as-fed basis. The feed-to-gain ratio was calculated for each sheep by dividing ADFI by ADG, and the individual ratios were then averaged within each group.

On day 50, after a 24 h feed withdrawal with free access to water, 4 mL of jugular blood was collected from each sheep at 10:00 before morning feeding into a red-cap clot-activator tube. Blood was allowed to clot for 8 h at room temperature and was then centrifuged at 4000 rpm at 20 °C for 30 min in an IKEME ME6050 centrifuge (Guangzhou Wei’ai Health Technology Co., Ltd., Guangzhou, China); serum was stored at −20 °C. Total protein (TP), albumin (ALB), globulin (GLB), total cholesterol (TC), high-density lipoprotein cholesterol (HDL), aspartate aminotransferase (AST), alanine aminotransferase (ALT), and γ-glutamyl transferase (γ-GT) were analyzed colorimetrically on a Mindray BS-420 automated biochemical analyzer with reagents from Zhongsheng Beikong Biotechnology Co., Ltd. (Beijing, China). Tumor necrosis factor α (TNF-α), interleukin-6 (IL-6), and interleukin-4 (IL-4) were measured with colorimetric enzyme-linked immunosorbent assay kits from the Beijing Huaying Institute of Biological Technology (Beijing, China). Malondialdehyde (MDA), total antioxidant capacity (T-AOC), glutathione peroxidase (GSH-Px), and superoxide dismutase (SOD) were measured with colorimetric assay kits from the same manufacturer. Absorbance was measured on a DR-200BS microplate analyzer (Wuxi Huawei Diatek Instrument Co., Ltd., Wuxi, China). Catalog and lot numbers for the biochemical reagents and assay kits were not retained in the archived study records.

### 2.4. Duodenal Histology

After slaughter, a 1–2 cm duodenal segment was collected from each sheep approximately 10 cm distal to the pylorus. Samples were fixed for 48 h in 4% neutral paraformaldehyde (Servicebio, G1101; Wuhan, China), paraffin-embedded, cut at 5 μm on an RM2016 microtome (Leica, Shanghai, China), and H&E-stained with a Servicebio G1076 kit. Eight sections per sheep were examined with a Nikon ECLIPSE E100 upright optical microscope and DS-U3 imaging system (Nikon, Tokyo, Japan). Following a low-magnification survey of these sections, five nonoverlapping fields per sheep were selected systematically along intact mucosa at 100× total magnification. Saiviewer software (version 3.0.1) was used for digital observation and measurement of villus height, crypt depth, mucosal thickness, and muscular layer thickness. Ten longitudinally oriented, intact villus–crypt units with visible villus tips and crypt bases were selected per sheep. Structures that were sectioned obliquely or transversely, folded, damaged, or incompletely oriented were excluded. All sections were evaluated by an observer blinded to treatment, and the mean for each sheep was used as the experimental unit.

### 2.5. Untargeted Metabolomics of Duodenal Contents

Duodenal contents from all sheep were collected following slaughter, snap-frozen in liquid nitrogen, and stored at −80 °C. Untargeted metabolomics was performed by BGI (project F25A040011631; execution order W251201017A). For each sample, 25 mg of duodenal content was mixed with 10 μL of internal-standard solution and 800 μL of methanol/acetonitrile/water (2:2:1, *v*/*v*/*v*), ground for 5 min, kept at −20 °C for 2 h, and centrifuged at 25,000× *g* at 4 °C for 15 min. A 600-μL aliquot of the supernatant was freeze-dried, reconstituted in 250 μL of 50% methanol, and centrifuged again under the same conditions. A pooled quality-control (QC) sample was prepared by combining a 30-μL aliquot of supernatant from each sample. Chromatographic separation was performed on an ACQUITY UPLC I-Class PLUS system (Waters, Milford, MA, USA) fitted with a Waters BEH C18 column (2.1 × 100 mm, 1.7 μm; Waters, USA). In positive-ion mode, mobile phases A and B were water and methanol, respectively, each containing 0.1% formic acid. In negative-ion mode, mobile phases A and B were water and 95% methanol, respectively, each containing 10 mM ammonium formate. The gradient was maintained at 2% B from 0 to 1 min, increased from 2 to 98% B from 1 to 6 min, held at 98% B from 6 to 8 min, decreased to 2% B from 8 to 8.1 min, and then maintained at 2% B from 8.1 to 10 min. The flow rate, column temperature, and injection volume were 0.35 mL/min, 45 °C, and 5 μL, respectively.

MS1 and MS2 data were acquired on an Orbitrap Exploris 480 mass spectrometer (Thermo Fisher Scientific, Cambridge, MA, USA) with electrospray ionization in positive- and negative-ion modes. The scan ranges in positive- and negative-ion modes were m/z 70–1050 and 92–1050, respectively. For MS1, the resolution was 120,000, the automatic gain control (AGC) target was 3 × 10^6^, and the maximum injection time was 100 ms. The three most intense precursor ions in each scan were selected for fragmentation. For MS2, the resolution was 30,000, AGC target was 1 × 10^5^, maximum injection time was 50 ms, and stepped normalized collision energies were 20, 40, and 60. The sheath- and auxiliary-gas settings were 40 and 10, respectively; spray voltages were 3.80 kV in positive-ion mode and 3.20 kV in negative-ion mode; and the ion-transfer-tube and auxiliary-gas-heater temperatures were 320 and 350 °C, respectively. Raw data were processed in Compound Discoverer version 3.3 (Thermo Fisher Scientific, USA), with annotation against the BGI Metabolome Database, mzCloud, and ChemSpider. The precursor-ion, fragment-ion, and retention-time tolerances were <5 ppm, <10 ppm, and <0.2 min, respectively. Data were normalized by probabilistic quotient normalization in metaX, corrected using QC-based robust locally estimated scatterplot smoothing signal correction, and filtered to retain features with a coefficient of variation (CV) ≤ 30% in QC samples. Quality was assessed from base-peak chromatogram overlap, tight clustering of QC samples in principal component analysis (PCA), and correlations among pooled QC replicates. Partial least-squares discriminant analysis (PLS-DA) was used to derive VIP scores and visualize group separation; the score plot was treated as descriptive, and no claim of model significance was based on visual separation. Nominal differential liquid chromatography–mass spectrometry (LC–MS) features were screened using variable importance in projection (VIP) ≥ 1, fold change ≥ 1.2 or ≤0.83, and a two-sided Student’s *t*-test with an unadjusted threshold of *p* < 0.05. Benjamini–Hochberg-adjusted q values were used to account for multiple testing, with q < 0.05 defining statistical significance after false-discovery-rate correction. Kyoto Encyclopedia of Genes and Genomes (KEGG) enrichment was performed on the database-annotated nominal feature set and was considered exploratory because only one annotated feature met the significance level of q < 0.05.

### 2.6. Liver Transcriptomics

Liver tissue was collected immediately after slaughter, snap-frozen in liquid nitrogen, and stored at −80 °C until further analysis. Following RNA extraction and cDNA library construction, reads were filtered with SOAPnuke version 2.3.2 and aligned to the *Ovis aries* reference genome (NCBI GCF_016772045.2, ARS-UI_Ramb_v3.0) with HISAT2 version 2.2.1 [20]. Gene-set alignment was performed with Bowtie2 version 2.4.5 [21], and transcript abundance was estimated with RSEM version 1.3.1 [22].

Differential expression was analyzed with DEGseq version 1.54.0 [23]; genes with absolute log_2_FC ≥ 1 and q ≤ 0.001 were considered differentially expressed. Gene Ontology (GO) and KEGG enrichment analyses were performed using hypergeometric tests after correction for false discovery rate, and q ≤ 0.05 was considered significant [24]. Differential alternative splicing was analyzed from paired-end data with rMATS version 4.1.2 [25]. Skipped-exon events with a false-discovery rate < 0.05 were considered significant; no additional change-in-percent-spliced-in (ΔPSI) threshold was applied in the archived workflow. No qRT-PCR validation of selected differentially expressed genes was performed.

### 2.7. Statistical Analysis

Each sheep was considered an independent experimental unit (*n* = 6 per group). Growth, serum, and histomorphometric data were summarized as group means with pooled SEM. Analyses were performed in IBM SPSS Statistics version 26.0. Normality was assessed with the Shapiro–Wilk test, and homogeneity of variance was assessed with Levene’s test. Two-sided independent-samples *t*-tests assuming equal variances were used when the homogeneity assumption was met; Welch’s unequal-variance *t*-test was used when the homogeneity assumption was not met. For histomorphometric outcomes, the equivalent one-way analysis-of-variance statistic (F = t^2^) was also reported. Results were considered statistically significant at *p* < 0.05.

Omics analyses used the platform workflows and thresholds specified above. Phenotype–omics correlation analysis was not performed because a verified individual-level mapping across serum, metabolomic, and transcriptomic datasets was unavailable.

## 3. Results

### 3.1. Growth Performance

Average daily gain, average daily feed intake, and feed-to-gain ratio were not affected by PHL supplementation in Hu sheep (*p* > 0.05; Table 2).

### 3.2. Serum Biochemical Profile

Serum biochemical variables were not affected by PHL supplementation in Hu sheep (*p* > 0.05; Table 3).

### 3.3. Serum Immune and Antioxidant Responses

Serum IL-6 was lower and IL-4 was higher in the sheep supplemented with PHL than in the CON group (*p* < 0.001 and *p* = 0.024, respectively), whereas TNF-α did not differ significantly (*p* = 0.237; Table 4).

Serum MDA concentration was lower and GSH-Px activity was higher in the sheep supplemented with PHL than in the CON group (*p* = 0.003 and *p* < 0.001, respectively), whereas T-AOC and SOD were not affected by PHL supplementation (*p* > 0.05; Table 5).

### 3.4. Duodenal Histomorphometry

**Figure 1:**
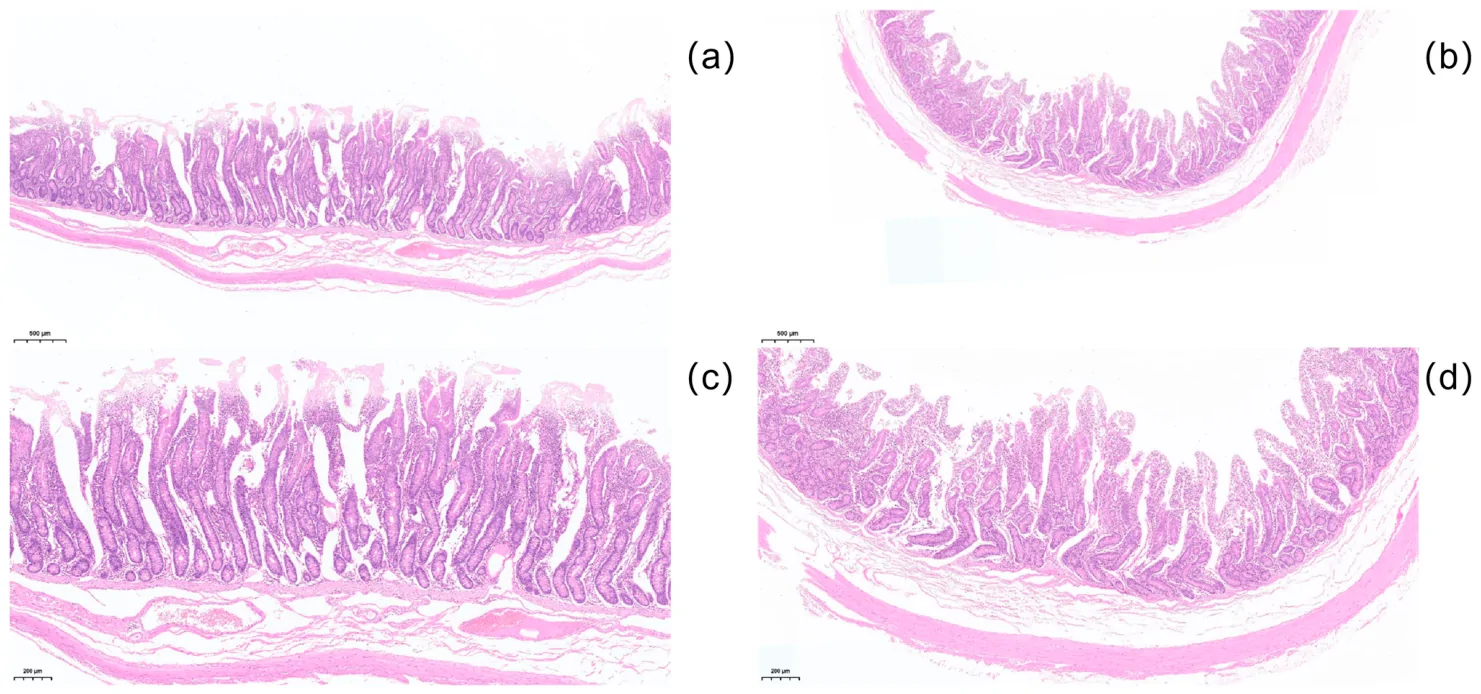
Hematoxylin and eosin (H&E)-stained duodenal sections. (**a**) Representative section from the control group (upper left); (**b**) representative section from the PHL group (upper right); (**c**) magnified view of panel (**a**) (lower left); and (**d**) magnified view of panel (**b**) (lower right).

The duodenal mucosa remained structurally intact in both groups, with identifiable villi, crypts, mucosa, and muscular layers. No obvious epithelial sloughing, necrosis, or severe tissue damage was observed in either group (Figure 1).

Villus height, crypt depth, and mucosal thickness were lower (*p* < 0.05) and muscular layer thickness was higher (*p* = 0.031; Table 6) in the sheep supplemented with PHL than in the CON group.

### 3.5. Quality Control and Global Features of Duodenal Metabolomics

Of the 53,716 detected LC–MS features, 32,275 met the CV ≤ 30% quality criterion (60.08%). Among the retained features, 5603 had database annotations and 26,672 were unannotated (Table 7).

**Figure 2:**
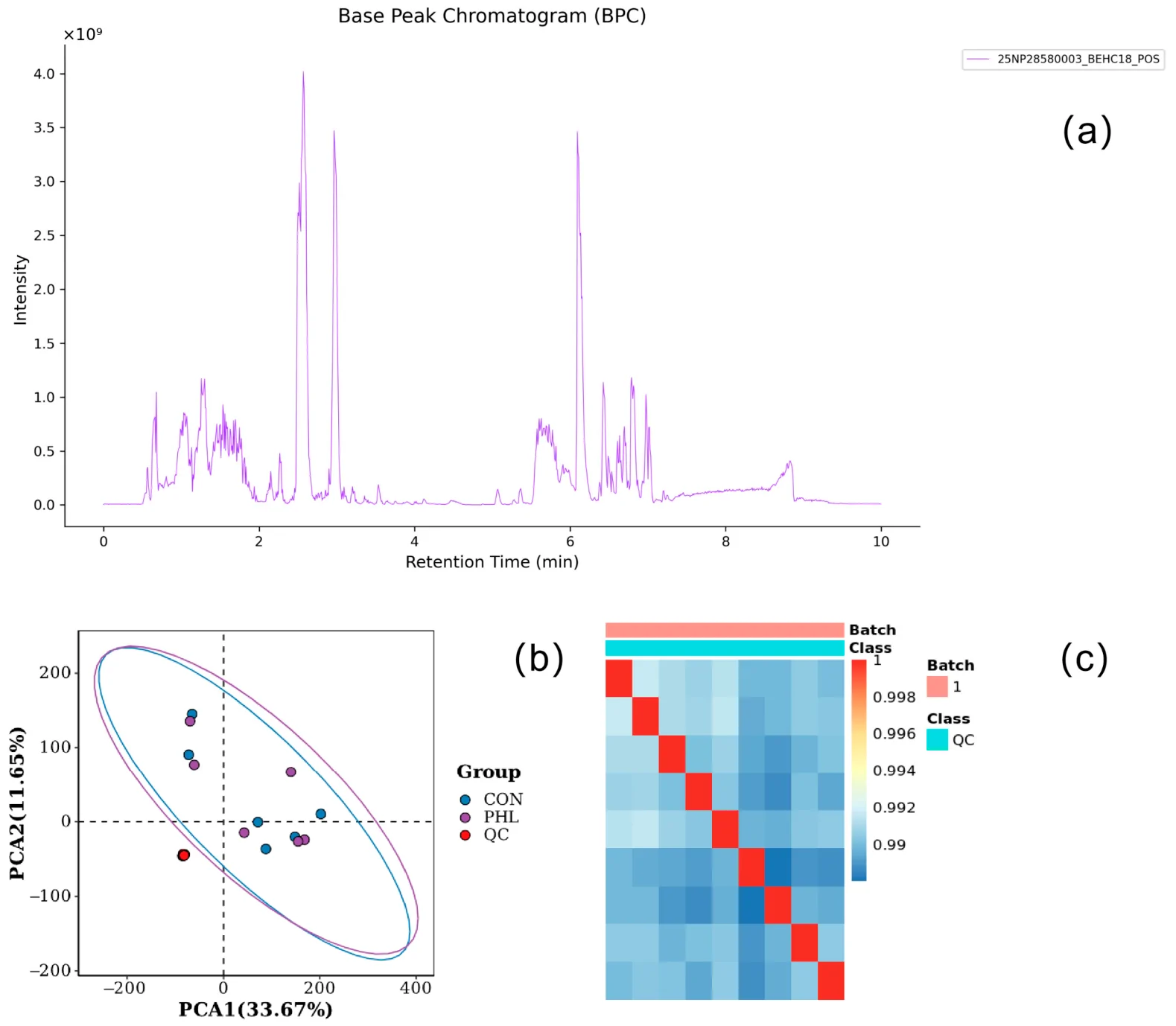
Quality-control assessment of duodenal untargeted metabolomics data. (**a**) Base-peak chromatogram (BPC) overlay; (**b**) principal component analysis (PCA) score plot of quality-control (QC) and biological samples; and (**c**) QC correlation heatmap.

Base-peak chromatograms overlapped closely, QC samples clustered tightly in PCA space, and correlations among QC replicates were high (Figure 2). Collectively, these QC results supported acceptable analytical repeatability; they were not used as evidence of biological separation between groups.

### 3.6. Nominal LC–MS Feature Screening and Exploratory Pathway Enrichment in the Duodenum

PCA showed partial separation between the groups, although considerable overlap existed. Because PCA is descriptive, this pattern alone was not considered evidence of statistically significant treatment effects. The PLS-DA score plot was likewise interpreted descriptively.

**Figure 3:**
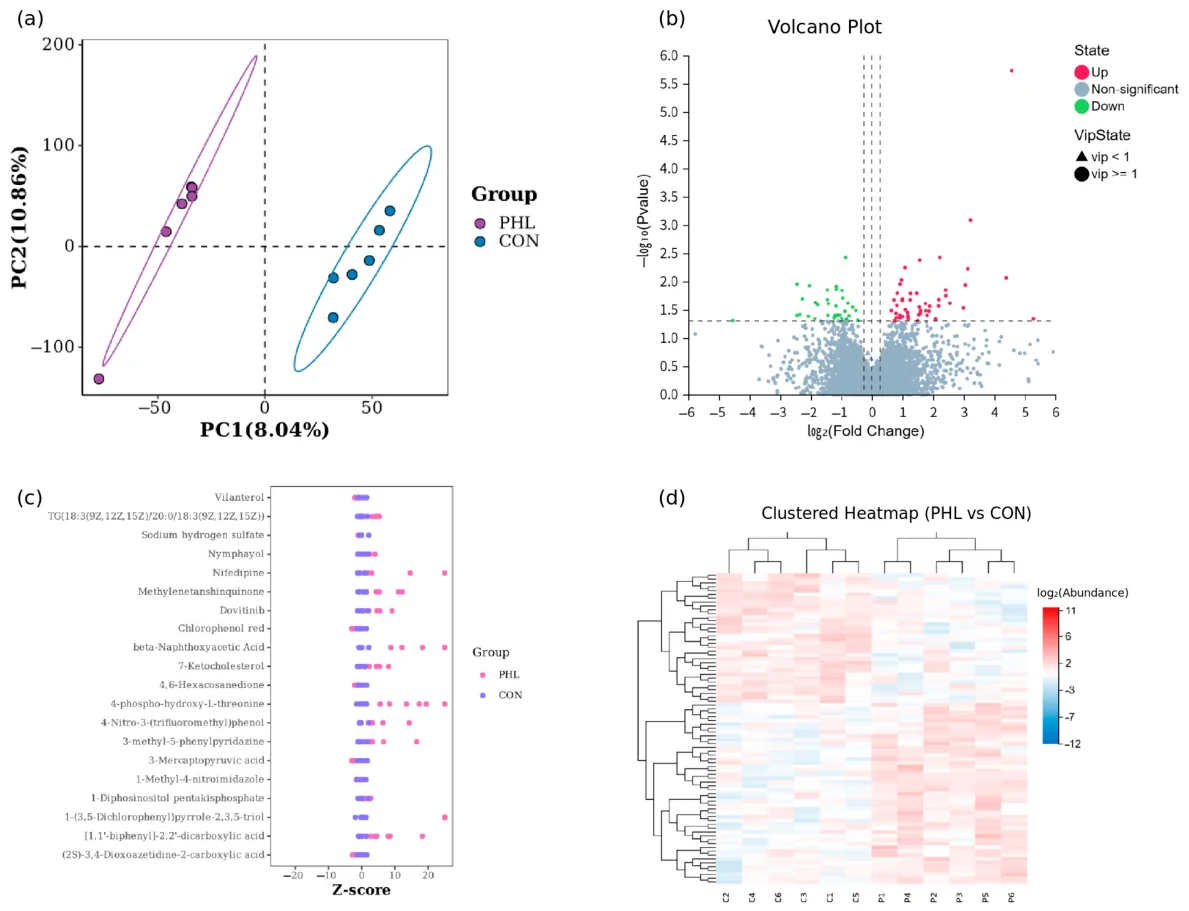
Profiling of database-annotated duodenal LC–MS features. Among 5603 annotated features, 81 met the nominal criteria of VIP ≥ 1, fold change ≥ 1.2 or ≤ 0.83, and unadjusted *p* < 0.05 (47 more abundant and 34 less abundant in the PHL group). (**a**) Partial least-squares discriminant analysis (PLS-DA) score plot, interpreted descriptively; (**b**) volcano plot; (**c**) Z-score plot; and (**d**) clustered heatmap. CON: control group; PHL: *P. harmala* stem ethanolic extract group.

Using VIP ≥ 1, fold change ≥ 1.2 or ≤0.83, and a two-sided *t*-test at an unadjusted threshold of *p* < 0.05, 479 LC–MS features met the nominal screening criteria; 270 were more abundant and 209 were less abundant in the sheep supplemented with PHL than in the CON group. Of these, 81 features had database annotations and 398 were unannotated. After Benjamini–Hochberg correction, two features met the criteria of q < 0.05, but only one was annotated. Thus, the 479-feature set should be considered an exploratory set of nominally significant features rather than a set of differential features confirmed after false discovery rate correction (Figure 3).

**Figure 4:**
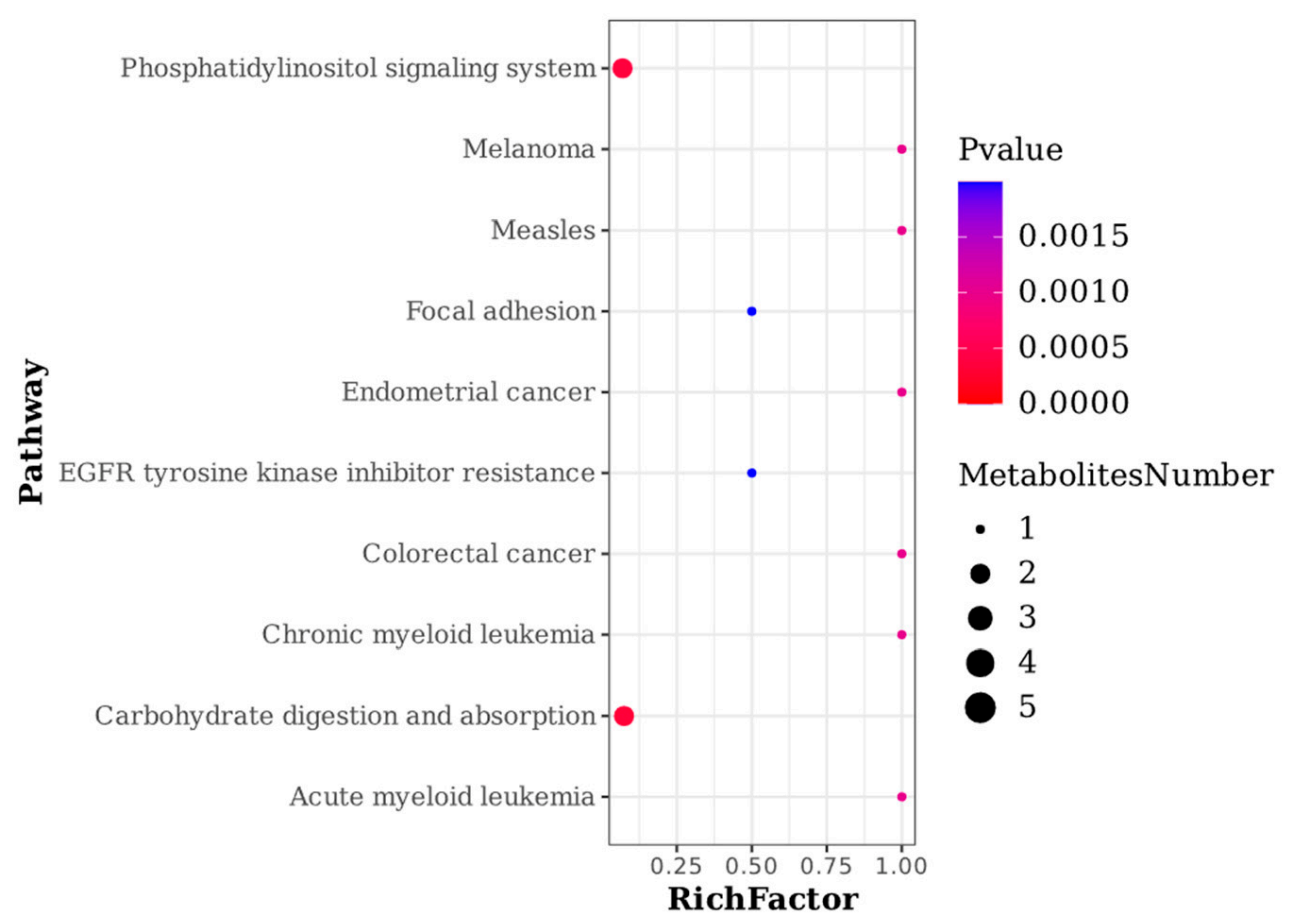
Exploratory KEGG pathway enrichment based on 20 KEGG-mapped features within the 81 database-annotated nominal LC–MS features in duodenal contents. Bubble color represents the unadjusted *p* value, and bubble size represents the number of mapped features.

Exploratory KEGG analysis was performed on the database-annotated nominal feature set. Twenty annotated features mapped to 79 pathways, with carbohydrate digestion and absorption and phosphatidylinositol signaling among the leading annotations (Figure 4). An LC–MS feature putatively annotated as α-lactose was nominally less abundant (fold change = 0.37, unadjusted *p* = 0.0245), whereas features putatively annotated as phosphatidylinositol-3,4,5-trisphosphate and 1-diphosphoinositol pentakisphosphate were nominally more abundant (fold changes = 1.89 and 1.90, respectively) in the sheep supplemented with PHL than in the CON group. These compound identities were not confirmed with authentic standards. Because the feature-level and pathway results were derived from the unadjusted screening set, they should be considered hypothesis-generating.

### 3.7. Liver Transcriptome Quality and Differential Expression

Each hepatic transcriptome library yielded an average of 6.62 Gb of clean bases. Q20 and Q30 ranged from 96.99% to 98.12% and from 90.68% to 93.69%, respectively. Clean-read retention ranged from 95.13% to 96.42%; the mean genome and gene-set mapping rates were 97.82% and 75.62%, respectively; and 18,489 genes were detected (Table 8).

**Figure 5:**
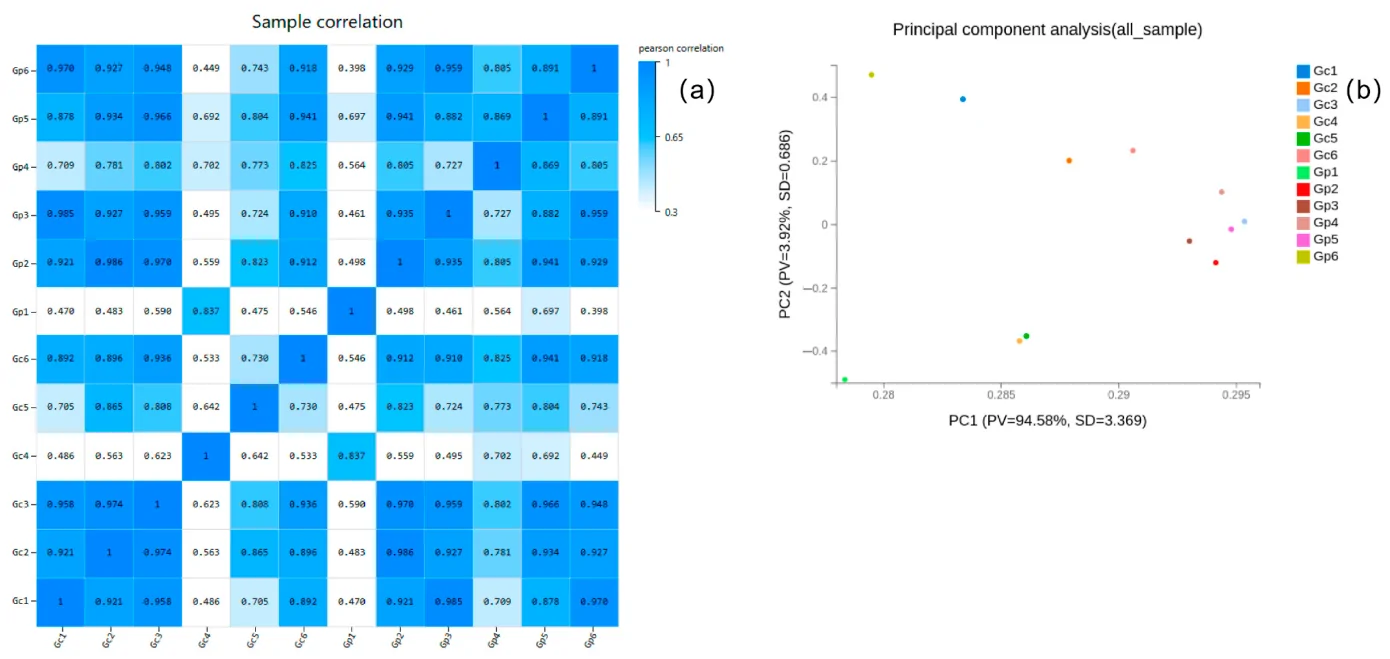
Quality assessment of liver transcriptomes from Hu sheep supplemented with or without PHL. (**a**) Sample correlation heatmap; (**b**) principal component analysis (PCA) score plot. In the figure, Gc1–Gc6 denote CON samples and Gp1–Gp6 denote PHL samples.

**Figure 6:**
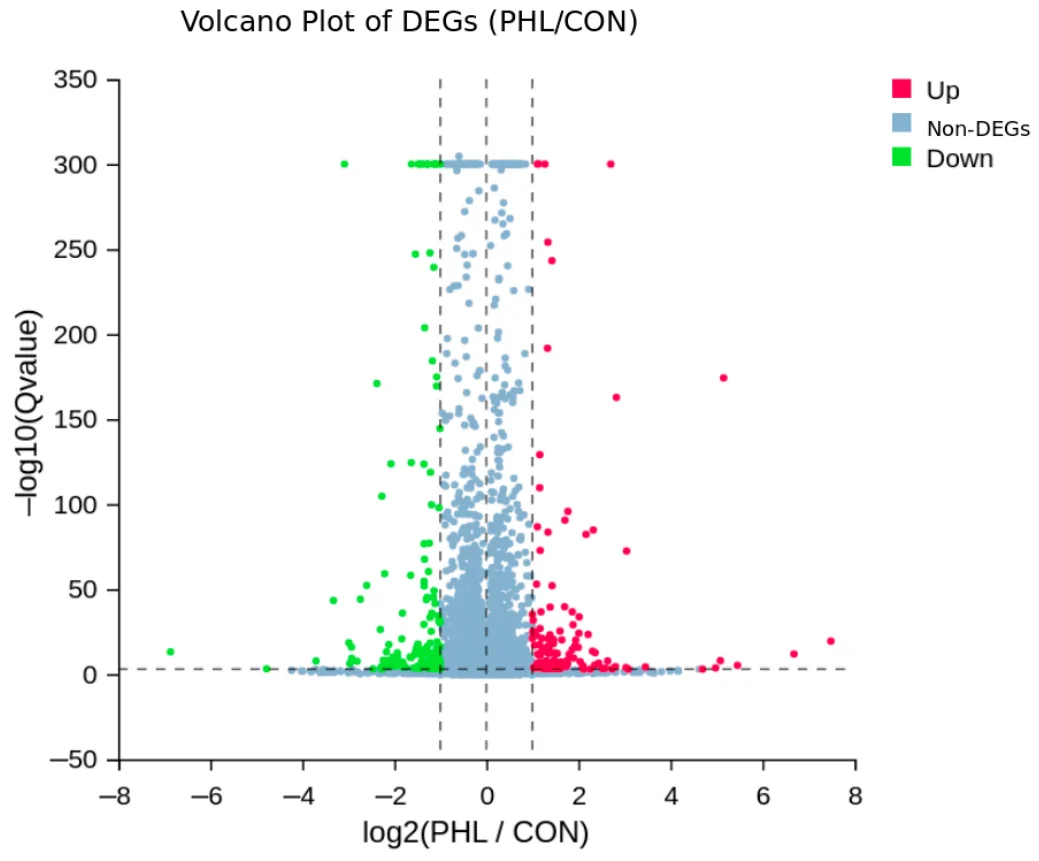
Volcano plot of genes meeting the predefined differential-expression criteria in liver transcriptomes of Hu sheep supplemented with or without PHL. Q value denotes the DEGseq multiple-testing-adjusted value. The vertical dashed lines indicate log_2_FC values of −1, 0, and 1, and the horizontal dashed line indicates q = 0.001. Genes with |log_2_FC| ≥ 1 and q ≤ 0.001 were considered differentially expressed.

Within-group correlations were high, and PCA showed partial separation between groups (Figure 5). Using DEGseq v1.54.0 with absolute log_2_FC ≥ 1 and q ≤ 0.001, 279 genes met the predefined RNA-seq differential-expression criteria; 135 were upregulated and 144 were downregulated in the PHL group (Figure 6).

Several genes showed marked expression changes. The upregulated genes included CYP1A1, ABCC11, HKDC1, PLA2G2F, TFF2, TFF3, ELOVL7, and ADIPOQ, whereas the downregulated genes included FASN, SLC2A5, SLC51A, MIOX, ANPEP, APOA4, ORM1, and TNC. The annotated functions of these genes encompassed transport, substrate use, detoxification, lipid metabolism, and tissue-interface processes in the liver.

### 3.8. Functional Enrichment and Alternative Splicing in the Liver

**Figure 7:**
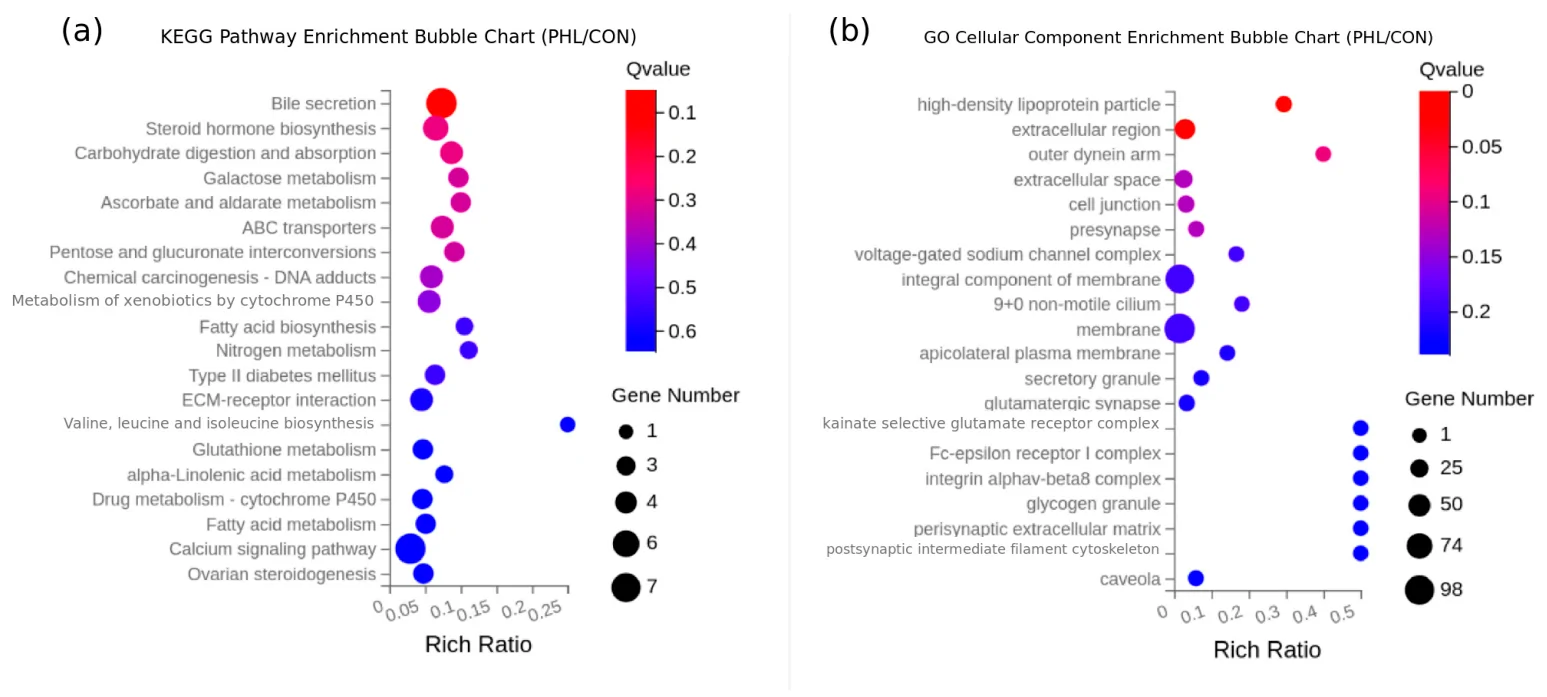
Functional enrichment analysis of genes meeting the predefined differential-expression criteria in the liver of Hu sheep supplemented with or without PHL. (**a**) KEGG pathway enrichment bubble plot; (**b**) GO cellular-component enrichment bubble plot. Bubble color represents q value, and bubble size represents gene number; pathway annotations are interpreted descriptively.

KEGG analysis highlighted several leading pathway annotations in the liver, including bile secretion, carbohydrate digestion and absorption, steroid hormone biosynthesis, ATP-binding cassette (ABC) transporters, ascorbate and aldarate metabolism, glutathione metabolism, fatty acid metabolism, and fatty acid biosynthesis (Figure 7a). SLC51A, ABCC11, and FXYD2 were represented in bile-acid and membrane-transport annotations; HKDC1 and SLC2A5 in carbohydrate-handling annotations; FASN and ELOVL·7 in lipid-metabolism annotations; and GGT6 and ANPEP in glutathione-related redox annotations.

GO analysis highlighted cellular-component terms including high-density lipoprotein particle, extracellular region, extracellular space, and cell-junction-related categories (Figure 7b). APOA4, ADIPOQ, CLDN7, CLDN8, SPP1, TNC, and DPEP1 were represented in pathway annotations and functional terms associated with lipoprotein transport, extracellular matrix organization, and membrane-interface regulation.

**Figure 8:**
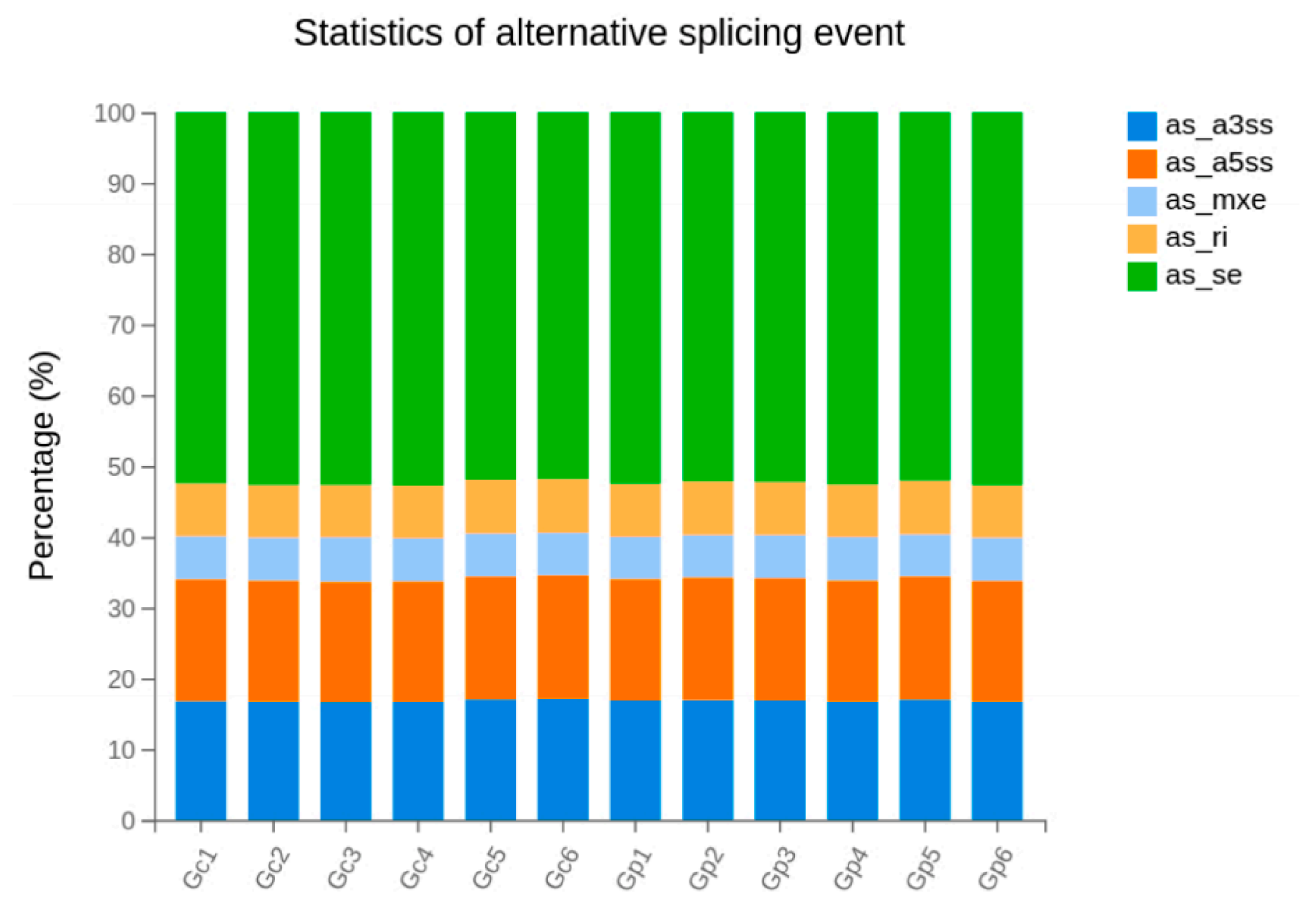
Distribution of alternative-splicing event types in liver transcriptome samples from Hu sheep. as_3ss: alternative 3′ splice site; as_5ss: alternative 5′ splice site; as_mxe: mutually exclusive exons; as_ri: retained intron; as_se: skipped exon.

Alternative splicing analysis detected 52,079 events, of which skipped-exon events were the predominant type (Figure 8). Among 5576 candidate skipped-exon events, 104 events involving 92 genes had a false-discovery rate < 0.05. Representative genes included TSC22D1, ADAMTS13, RAD51B, B4GALT2, PLXNB2, MVK, and RBM4.

### 3.9. Cross-Tissue Summary of Measured Responses

The nominal, database-annotated duodenal feature set yielded exploratory pathway annotations involving carbohydrate digestion and absorption and phosphatidylinositol signaling. In the liver, DEG-level evidence was controlled at q ≤ 0.001, whereas pathway annotations were interpreted descriptively; leading annotations involved bile secretion, ABC-mediated transport, carbohydrate and lipid metabolism, and glutathione-related redox functions.

The hepatic transcriptome therefore provided false-discovery-rate-controlled evidence at the gene and splice-event levels in this trial. The nominal duodenal LC–MS feature results may serve as a basis for targeted validation but do not establish a causal intestinal mechanism.

## 4. Discussion

### 4.1. Animal-Level Responses to PHL Supplementation

IL-6 and MDA were lower and IL-4 and GSH-Px were higher in the sheep supplemented with PHL than in the CON group. In contrast, feed intake, ADG, feed efficiency, and routine serum biochemical variables were not affected by PHL treatment.

Meta-analyses of ruminant flavonoid supplementation have indicated heterogeneous performance responses [15,16]. The absence of significant growth effects in the present study may reflect the small cohort, 50-day duration, single 3 g extract/head/day dose, delivered total-flavonoid amount (approximately 197.1 mg RE/head/day; planned target: 200 mg RE/head/day), or differences in extract composition, breed, basal diet, and management. Because no dose–response study of *P. harmala* was available, the selected dose was supported only indirectly by studies of other ruminant plant or flavonoid preparations [15,16,17,18,19].

Routine serum biochemical variables, including AST, ALT, and γ-GT, were not affected by PHL treatment. The absence of statistically significant differences may reflect the dose, 50-day exposure, small group size, or limited systemic exposure to active extract constituents; unchanged enzyme activities do not by themselves establish safety. TP, ALB, and GLB did not differ significantly between groups, so these data do not establish altered protein metabolism or humoral immunity due to PHL supplementation. The combination of lower IL-6 and higher IL-4 was directionally consistent with reported anti-inflammatory actions of flavonoids and *P. harmala* extracts [4,9], but it reflected only the limited cytokine panel measured in this study. However, TNF-α was unaffected, and the three measured cytokines were insufficient to define an immune mechanism. Lower MDA indicated reduced accumulation of lipid-peroxidation products, whereas higher GSH-Px activity indicated greater enzymatic peroxide-removal capacity. T-AOC and SOD were unaffected by PHL treatment in the present trial.

### 4.2. Tissue-Level and Omics Evidence

Quantitative duodenal histomorphometry showed lower villus height, crypt depth, and mucosal thickness together with greater muscular layer thickness in the sheep supplemented with PHL than in the CON group. The total-flavonoid values for the raw material and the administered product must be distinguished: the dried stems contained 10.67 mg RE/g, whereas the vacuum-dried concentrated extract fed to the sheep contained 65.7 mg RE/g, representing a 6.16-fold enrichment and providing approximately 197.1 mg RE/head/day of total flavonoids from 3 g extract/head/day. This exposure description establishes that the sheep received a concentrated, multi-constituent ethanolic extract rather than raw stem material; it does not identify which constituent or constituent class was responsible for the histomorphometric differences. In the present study, the concurrent reductions in villus height and crypt depth should not be classified as either beneficial or adverse without functional measurements. No obvious epithelial sloughing, necrosis, or severe tissue damage was observed in any treatment group. Moreover, because PHL was not a purified flavonoid fraction and exploratory profiling tentatively detected co-extracted alkaloids and phenolic acids, flavonoids cannot be assigned as the sole causal constituents. Therefore, intestinal permeability, digestive function, nutrient utilization, and constituent-specific exposure should be measured in future studies.

The metabolomic evidence requires strict qualification. Although 479 LC–MS features met the VIP, fold-change, and unadjusted *p*-value criteria, only 81 had database annotations. Two features met the Benjamini–Hochberg q < 0.05 threshold, and only one of these was annotated. Pathway interpretation still depends on annotation quality and appropriate correction for multiple testing. Here, the apparent carbohydrate-digestion and phosphatidylinositol-signaling enrichments were calculated from the annotated nominal set and could therefore include false-positive associations. These findings should be treated as hypotheses for targeted metabolite assays rather than as confirmed duodenal mechanisms.

The hepatic DEG analysis used a stringent threshold and identified 279 treatment-associated expression differences. Functional annotation highlighted bile secretion, ABC transport, nutrient metabolism, and glutathione-related categories. GGT6 and ANPEP contributed to glutathione-related annotations, and this transcriptional pattern was functionally concordant with higher serum GSH-Px activity and lower MDA concentration. These RNA-seq findings were not independently validated by qRT-PCR and should therefore be interpreted as sequencing-based, exploratory evidence. These functional annotations do not establish a mechanism for PHL. Transport genes such as SLC51A and ABCC11 were associated with the hepatic handling of endogenous or dietary compounds; pathway-level correspondence alone does not demonstrate that the transcriptional changes caused the serum phenotype. The 104 skipped-exon events that remained significant after false discovery rate correction provide further evidence of treatment-associated differences in transcript structure but require isoform-specific validation.

Individual-level mappings needed to correlate serum markers with metabolite abundance or hepatic transcripts were not available, so no phenotype–omics correlation analysis was performed. Consequently, cross-tissue links are based on functional concordance only. The data support treatment-associated differences in measured serum markers and hepatic molecular profiles, but not a single gut–liver causal pathway.

### 4.3. Limitations and Overall Interpretation

This study was limited by the inclusion of six sheep per group, one dose, and a 50-day exposure. The administered concentrated ethanolic extract had a total flavonoid content of 65.7 mg RE/g; harmine, tetrahydroharmol, deoxypeganine, vasicinone, 4(3H)-quinazolinone, several phenolic acids, and individual flavonoids were tentatively annotated by untargeted LC–MS but were not quantified using authentic standards; harmaline was not quantified, and no independent safety or dose-finding trial was conducted. The absence of feed refusal, disease, abnormal behavior, death, or routine serum biochemical abnormalities during the feeding period is an observation rather than proof of safety. The histomorphometric changes were not accompanied by measurements of intestinal permeability, digestive function, or nutrient utilization; only one database-annotated metabolic feature met q < 0.05; PLS-DA visualization was interpreted descriptively rather than as evidence of model significance; phenotype–omics correlations were unavailable; selected differentially expressed genes were not independently validated by qRT-PCR; and key splice events were not validated by isoform-specific assays. These limitations preclude treating individual gene-level changes as independently confirmed effects.

Future studies should include larger cohorts and multiple doses, quantify β-carboline alkaloids and individual flavonoids in each feeding batch, validate candidate metabolites with targeted assays, confirm selected differentially expressed genes by qRT-PCR, validate splice isoforms using isoform-specific assays, and preserve individual sample mapping for cross-tissue correlation analyses.

## 5. Conclusions

Supplementation with 3 g/head/day of *P. harmala* stem ethanolic extract (providing approximately 197.1 mg RE/head/day of total flavonoids) for 50 days was associated with lower serum IL-6 and MDA and higher IL-4 and GSH-Px in Hu sheep, without significant changes in growth performance or routine serum biochemistry. Hepatic RNA sequencing identified 279 genes meeting the predefined differential-expression criteria and 104 skipped-exon events meeting the criterion of false discovery rate < 0.05. Functional annotations included transport, nutrient metabolism, and redox-related categories. In contrast, 479 duodenal LC–MS features met nominal, unadjusted screening criteria, but only 81 were database-annotated and only one database-annotated feature remained significant at q < 0.05; therefore, the metabolomic pathway results are exploratory. These findings support targeted dose–response studies, batch-specific alkaloid and flavonoid quantification, and independent validation, including qRT-PCR confirmation of selected hepatic genes, before practical application.

## Figures and Tables

**Table 1:** Ingredient composition and nutrient contents of the basal complete pelleted diet for Hu sheep.

Ingredient	Proportion (%)	Nutrient	Content
Corn	49.85	DM (% as-fed)	88.6
Soybean meal	12.00	ME (MJ/kg DM)	11.7
Cottonseed meal	20.00	CP (% DM)	20.9
Wheat bran	10.00	NDF (% DM)	18.3
Sodium bicarbonate	1.00	ADF (% DM)	8.0
Dicalcium phosphate	1.00	EE (% DM)	3.9
Salt	1.00	Starch (% DM)	39.2
Vitamin and mineral premix	5.00	Ca (% DM)	1.25
Mycotoxin binder	0.15	P (% DM)	0.80
Total	100.00	Na (% DM)	0.77

Note: Ingredient proportions are expressed on an air-dry basis. Dry-matter (DM) content was determined from the as-fed diet; metabolizable energy (ME), crude protein (CP), neutral detergent fiber (NDF), acid detergent fiber (ADF), ether extract (EE), starch, calcium (Ca), phosphorus (P), and sodium (Na) were calculated on a DM basis. Each kg of premix supplied vitamin A 4000 IU, vitamin D_3_ 800 IU, vitamin E 20 mg, Fe 15 mg, Cu 2 mg, Mn 10 mg, Zn 20 mg, Co 0.10 mg, I 0.50 mg, and Se 0.20 mg.

**Table 2:** Effects of PHL supplementation on growth performance in Hu sheep.

Parameter	Treatments		SEM	*p*-Value
	CON	PHL		
Initial body weight (kg)	33.21	33.12	0.60	0.924
Final body weight (kg)	43.63	44.99	0.41	0.056
Average daily gain (g/d)	208.50	237.33	9.71	0.062
Average daily feed intake (g as-fed/d)	1311.04	1353.09	36.45	0.434
Feed-to-gain ratio	6.38	5.74	0.35	0.237

CON: control group; PHL: *P. harmala* stem ethanolic extract group; SEM: pooled standard error of the mean.

**Table 3:** Effects of PHL supplementation on serum biochemical parameters in Hu sheep.

Parameter	Treatments		SEM	*p*-Value
	CON	PHL		
TP (g/L)	67.58	70.69	1.88	0.268
ALB (g/L)	31.48	30.92	0.69	0.583
GLB (g/L)	36.11	39.77	1.83	0.186
TC (mmol/L)	1.18	1.09	0.10	0.520
HDL (mmol/L)	0.76	0.72	0.07	0.616
AST (U/L)	108.16	105.20	9.99	0.838
ALT (U/L)	16.15	12.86	1.73	0.209
γ-GT (U/L)	67.96	67.56	4.23	0.948

CON: control group; PHL: *P. harmala* stem ethanolic extract group; SEM: pooled standard error of the mean; TP: total protein; ALB: albumin; GLB: globulin; TC: total cholesterol; HDL: high-density lipoprotein cholesterol; AST: aspartate aminotransferase; ALT: alanine aminotransferase; γ-GT: γ-glutamyl transferase.

**Table 4:** Effects of PHL supplementation on serum inflammatory markers in Hu sheep.

Parameter	Treatments		SEM	*p*-Value
	CON	PHL		
TNF-α (pg/mL)	56.74	51.26	2.98	0.237
IL-6 (pg/mL)	124.06 ^a^	94.77 ^b^	2.63	<0.001
IL-4 (pg/mL)	5.67 ^b^	6.29 ^a^	0.16	0.024

CON: control group; PHL: *P. harmala* stem ethanolic extract group; SEM: pooled standard error of the mean; TNF-α: tumor necrosis factor α; IL: interleukin. ^a,b^ Means with different superscripts within a row differ significantly (*p* < 0.05).

**Table 5:** Effects of PHL supplementation on serum antioxidant markers in Hu sheep.

Parameter	Treatments		SEM	*p*-Value
	CON	PHL		
MDA (mmol/L)	2.03 ^a^	1.73 ^b^	0.06	0.003
T-AOC (mmol/L)	0.59	0.61	0.02	0.378
GSH-Px (U/mL)	301.85 ^b^	393.19 ^a^	5.61	<0.001
SOD (U/mL)	9.04	9.41	0.29	0.398

CON: control group; PHL: *P. harmala* stem ethanolic extract group; SEM: pooled standard error of the mean; MDA: malondialdehyde; T-AOC: total antioxidant capacity; GSH-Px: glutathione peroxidase; SOD: superoxide dismutase. ^a,b^ Means with different superscripts within a row differ significantly (*p* < 0.05).

**Table 6:** Effects of PHL supplementation on duodenal histomorphometric measurements in Hu sheep.

Parameter	Treatments		F-Value	SEM	*p*-Value
	CON	PHL			
Villus height (μm)	660.36 ^a^	563.27 ^b^	8.867	23.06	0.014
Crypt depth (μm)	265.45 ^a^	218.18 ^b^	9.219	11.01	0.013
Mucosal thickness (μm)	925.82 ^a^	781.45 ^b^	19.782	22.95	0.001
Muscular layer thickness (μm)	128.73 ^b^	140.36 ^a^	6.315	3.27	0.031

CON: control group; PHL: *P. harmala* stem ethanolic extract group; SEM: pooled standard error of the mean; F: test statistic from the equivalent two-group one-way analysis of variance (F = t^2^). ^a,b^ Means with different superscripts within a row differ significantly (*p* < 0.05).

**Table 7:** Summary of quality-control metrics and differential features in duodenal untargeted metabolomics data from Hu sheep.

Item	Value
Total LC–MS features detected	53,716
LC–MS features with CV ≤ 30%	32,275
Proportion with CV ≤ 30%	60.08%
LC–MS features significant after false discovery rate correction (total/annotated; q < 0.05)	2/1
Database-annotated LC–MS features	5603
Nominal differential LC–MS features (all; unadjusted *p* < 0.05)	479
Nominally higher LC–MS features	270
Nominally lower LC–MS features	209
KEGG-mapped annotated nominal features	20
Pathways in exploratory KEGG analysis	79

**Table 8:** Summary of liver transcriptome sequencing quality in Hu sheep.

Item	Value
Number of samples	12
Average clean bases (Gb)	6.62
Q20 range (%)	96.99–98.12
Q30 range (%)	90.68–93.69
Clean-read retention (%)	95.13–96.42
Average genome mapping rate (%)	97.82
Average gene-set mapping rate (%)	75.62
Genes detected	18,489

## Data Availability

The data presented in this study are available from the corresponding authors upon reasonable request.

## References

[1] Wang J., Deng L., Chen M., Che Y., Li L., Zhu L., Chen G., Feng T. (2024). Phytogenic feed additives as natural antibiotic alternatives in animal health and production: A review of the literature of the last decade. Anim. Nutr..

[2] Nastoh N.A., Waqas M., Çınar A.A., Salman M. (2024). The impact of phytogenic feed additives on ruminant production: A review. J. Anim. Feed. Sci..

[3] Biswas S., Kim I.H. (2025). A thorough review of phytogenic feed additives in non-ruminant nutrition: Production, gut health, and environmental concerns. J. Anim. Sci. Technol..

[4] Chen S., Wang X., Cheng Y., Gao H., Chen X. (2023). A review of classification, biosynthesis, biological activities and potential applications of flavonoids. Molecules.

[5] Shen N., Wang T., Gan Q., Liu S., Wang L., Jin B. (2022). Plant flavonoids: Classification, distribution, biosynthesis, and antioxidant activity. Food Chem..

[6] Li L.-N. (2024). Peganum harmala L.: A review of botany, traditional use, phytochemistry, pharmacology, quality marker, and toxicity. Comb. Chem. High Throughput Screen..

[7] Sharifi-Rad J., Quispe C., Herrera-Bravo J., Semwal P., Painuli S., Özçelik B., Hacıhasanoğlu F.E., Shaheen S., Sen S., Acharya K. (2021). *Peganum* spp.: A comprehensive review on bioactivities and health-enhancing effects and their poten-tial for the formulation of functional foods and pharmaceutical drugsOxidative. Med. Cell. Longev..

[8] İzol E., Turhan M., Yapıcı İ., Necip A., Yılmaz M.A., Zengin G. (2025). Research of Peganum harmala: Phytochemical content, mineral profile, antioxidant, antidiabetic, anticholinergic properties, and molecular docking. Chem. Biodivers..

[9] Abbas M.W., Hussain M., Qamar M., Ali S., Shafiq Z., Wilairatana P., Mubarak M.S. (2021). Antioxidant and anti-inflammatory effects of Peganum harmala extracts: An in vitro and in vivo study. Molecules.

[10] Chen L., Cao H., Huang Q., Xiao J., Teng H. (2022). Absorption, metabolism and bioavailability of flavonoids: A review. Crit. Rev. Food Sci. Nutr..

[11] Zhou M., Ma J., Kang M., Tang W., Xia S., Yin J., Yin Y. (2024). Flavonoids, gut microbiota, and host lipid metabolism. Eng. Life Sci..

[12] Baky M.H., Elshahed M., Wessjohann L., Farag M.A. (2022). Interactions between dietary flavonoids and the gut microbiome: A comprehensive review. Br. J. Nutr..

[13] Xiong H.-H., Lin S.-Y., Chen L.-L., Ouyang K.-H., Wang W.-J. (2023). The interaction between flavonoids and intestinal microbes: A review. Foods.

[14] Piao M., Tu Y., Zhang N., Diao Q., Bi Y. (2023). Advances in the application of phytogenic extracts as antioxidants and their potential mechanisms in ruminants. Antioxidants.

[15] Lucio-Ruíz F., Godina-Rodríguez J.E., Granados-Rivera L.D., Orzuna-Orzuna J.F., Joaquín-Cancino S., Hernández-García P.A. (2024). Meta-analysis of dietary supplementation with flavonoids in small ruminants: Growth performance, antioxidant status, nutrient digestibility, ruminal fermentation, and meat quality. Small Rumin. Res..

[16] Orzuna-Orzuna J.F., Dorantes-Iturbide G., Lara-Bueno A., Chay-Canul A.J., Miranda-Romero L.A., Mendoza-Martínez G.D. (2023). Meta-analysis of flavonoids use into beef and dairy cattle diet: Performance, antioxidant status, ruminal fermentation, meat quality, and milk composition. Front. Vet. Sci..

[17] Yu S., Li L., Zhao H., Zhang S., Tu Y., Liu M., Zhao Y., Jiang L. (2023). Dietary citrus flavonoid extract improves lactational performance through modulating rumen microbiome and metabolites in dairy cows. Food Funct..

[18] Li M., Hassan F., Peng L., Xie H., Liang X., Huang J., Huang F., Guo Y., Yang C. (2022). Mulberry flavonoids modulate rumen bacteria to alter fermentation kinetics in water buffalo. PeerJ.

[19] Du J., Wang Y., Su S., Wang W., Guo T., Hu Y., Yin N., An X., Qi J., Xu X. (2025). Supplementation with complex phytonutrients enhances rumen barrier function and growth performance of lambs by regulating rumen microbiome and metabolome. Animals.

[20] Kim D., Paggi J.M., Park C., Bennett C., Salzberg S.L. (2019). Graph-based genome alignment and genotyping with HISAT2 and HISAT-genotype. Nat. Biotechnol..

[21] Langmead B., Salzberg S.L. (2012). Fast gapped-read alignment with Bowtie 2. Nat. Methods.

[22] Li B., Dewey C.N. (2011). RSEM: Accurate transcript quantification from RNA-Seq data with or without a reference genome. BMC Bioinform..

[23] Wang L., Feng Z., Wang X., Wang X., Zhang X. (2010). DEGseq: An R package for identifying differentially expressed genes from RNA-seq data. Bioinformatics.

[24] Kanehisa M., Furumichi M., Sato Y., Ishiguro-Watanabe M., Tanabe M. (2021). KEGG: Integrating viruses and cellular organisms. Nucleic Acids Res..

[25] Shen S., Park J.W., Lu Z.-X., Lin L., Henry M.D., Wu Y.N., Zhou Q., Xing Y. (2014). rMATS: Robust and flexible detection of differential alternative splicing from replicate RNA-Seq data. Proc. Natl. Acad. Sci. USA.

